# Clinical Notes from Private Practice

**Published:** 1849-10

**Authors:** George L. Upshur

**Affiliations:** Norfolk, Va., Member of the American Medical Association


### MEDICAL EXAMINER,

AND

### RECORD OF MEDICAL SCIENCE.

NEW SERIES.—NO. LVIII.—OCTOBER, 1849.

### ORIGINAL COMMUNICATIONS.

*Clinical Notes from Private Practice.* By GEORGE L. UPSHUR, M. D., of Norfolk, Va., Member of the American Medical Association.

*Dysentery and Miasmatic Fever.*—J. S., shoemaker, of sanguineous, lymphatic temperament, aged 28, married, was exposed to a heavy rain on the 8th of July, 1848, and got thoroughly wet. Was seized two days after with chill followed by dysentery—the discharges from the bowels amounting to ten in as many hours, and consisting almost entirely of blood. There was not much fever, but distressing tenesmus. I saw him on the 11th. He had taken a dose of castor oil the day before. Prescribed Hope's mixture, (Nitrous Acid ʒi., Tr. Op. gtt. xl., Aq. Camph. ℥viii)—afterwards Ol. Ricini and Tr. Op.—then sulphate of copper and opium, but with little or no abatement in the symptoms. Ten days after the commencement of the attack, he told me he thought he had had every afternoon, for several days past, a slight aching of the limbs and chilliness, and then he suffered most from his bowels. Prescribed Calomel gr. vi., Quiniæ Sulph. gr. xx., Morph. Sulph. gr. ½. M.

Pil. 8. S. one pill every two hours. Two days after, all the symptoms were improved, and he convalesced rapidly.

*Remarks.*—This case is a very good type of what we often meet with in lower Virginia. Persons are attacked by disease which seems to be purely local in its character, and is treated as such, but without much improvement. Upon a close examination, it will be discovered that the patient is much more unwell at one period of the day than at another—that he is more thirsty, and has some general aching of the limbs, which cannot be accounted for by the local disease. These symptoms are due to malarious influence—in other words, they are symptoms of ague. Under such circumstances you may treat the local disease *for ever*, without success. But if you will give the quinine freely, the local symptoms will disappear without further attention. I have seen this view frequently illustrated in this city during the summer and autumn. In fact, we rarely see an acute local disease that is not warped by this miasmatic agent.

*Typhoid Fever.*—This form of fever is rare in this section of country; so rare, that until the summer of 1848, I was disposed to doubt its existence here at all. I allude now, not to remittent fever which, sometimes runs into the typhoid or ataxic form, but to the true typhoid fever, or *dothinenterite*, of Louis and other French authors. Last summer I treated five well marked cases of this disease, and have seen three others since the first of last June. The rose-colored spots were found in five, epistaxis in one, and diarrhœa in all. The premonitory symptoms usually lasted ten days, and the disease was ushered in by rigors, followed by a tolerably high grade of fever, accompanied by headache and drowsiness. In one case, the gravest of all, the tongue remained *perfectly natural* throughout the disease. In every case there was a strong tendency to prostration. This want of energy was particularly noticed in the brain, in the form of drowsiness, low spirits, and listlessness, a condition of the great sensorium which it was extremely difficult to diagnose from that congestion of the brain, so often observed in typhoid and typhus fevers—especially as there was some heat of head and a flushed face during sleep. A stimulant such as Tr. Ammon. Arom. would arouse the patient in a few moments, and, so far from exciting the circulation, allayed it in a measure. The pulse, in every case, was very frequent, ranging from 110 to 130 per minute. This frequency of pulse continued some days, after all other unpleasant symptoms had disappeared, so that as long as it lasted, I felt sure the disease had not run its course, although the patient felt better;—and I could invariably predict, three or four days in advance, the approaching convalescence, simply from a lessening in the frequency of the pulse. The average duration of the disease was 21 days—the shortest being 15, and the longest 42 days.

The treatment consisted essentially of tonics from the very commencement—Tr. Cinchon. c., with small doses of quinine, alternating with port wine and wine of quassia, and now and then, in the latter stage of the disease, Dover's powder at night. The diarrhœa, in the earlier stage, was relieved by rhubarb and blue mass in small and frequently repeated doses, but would frequently recur towards the end of the second week, when strong astringents were called for. Of the eight cases, one died on the 42d day, being permitted to sink for want of proper attention on the part of the nurses.

*Remarks.*—Something useful may be deduced by us who practice in a malarious region, from a close study of such cases as those just alluded to. 1st. When a case is "beating up" for ten or twelve days, there being general malaise, whimsical appetite and unrefreshing sleep, it, in a majority of cases, turns out to be a case of typhoid fever. Therefore, when the patient takes to his bed and the doctor is sent for, he should be cautious not to prognosticate either a speedy or certain recovery. This caution is the more necessary in this region, where we are called to treat fevers chiefly of miasmatic origin, which are entirely amenable to proper treatment.

2nd. Do not use purgatives too freely. A drastic cathartic in the commencement of typhoid fever often complicates the whole subsequent treatment, by *fixing* the already strong tendency to the bowels, and thus starting a very ugly train of nervous symptoms.

3d. Do not become impatient at the length of time the fever lasts. These fevers seem to have a certain course to run—seldom less than 15 days—and it is therefore vain to attempt to *physic them* out of the system. All we can do is to watch symptoms—especially ataxic symptoms—which so frequently result from debility, and by treating them promptly and properly, ward off "the tendency to death." No man was ever in a better condition for having his stomach converted into an apothecary store. The most important thing to be learned in the practice of medicine, I believe, is, to know when *not* to give physic—*when* to let *well enough* alone. It must be confessed, however, that the *vis medicatrix naturæ* is too often, in the language of an eminent physician, "turned out of the room like a noisy cat." Homœopathy and Thompsonianism have taught the scientific physician two useful lessons; first, how often Nature is able to withstand the *disease;* and second, how often she is able to withstand the *doctor.* Typhoid fever, it must be remembered, is frequently as whimsical as a spoilt child, and requires fully as much *coaxing.*

*Laceration of the Perinæum.*—Mrs. W——, aged 32, of rigid fibre, was taken in labor with her first child, Sept. 6th, 1848. Saw her at 4 o'clock, A. M.—she had been suffering considerable pain since twelve the day before. Vertex presented in the 3d position of Baudelocque. At 10 o'clock was seized with pain across the forehead—stared wildly about—pulse labored, and only 52—pains active and recurring regularly every six minutes. Took twenty ounces of blood from the arm with entire relief to the head. At three o'clock, os uteri well dilated, but os externum and adjacent parts indisposed to yield; pains strong and frequent, but scarcely any progress made. I was called away at this time—returned in an hour—found the child expelled just as I entered the room, and the perinæum badly lacerated; I could not ascertain precisely to what extent, without an ocular examination, but from the touch I judged that it extended to the verge of the anus. The placenta was adherent to the right side of the uterus, about five inches from the os, and was delivered with difficulty.

The patient was directed to lie upon the side with the legs closed. On the 30th of September, the wound, not having been troubled in the mean time, was found to have healed by *first intention.* I was surprised at so favorable a result, especially as the patient, contrary to my directions, was out of bed on the third day.

*Prolapsus ani cured by operation.*—This is the case of a child two years old. The prolapsus, the result of cholera infantum, had existed two months. The mucous membrane was protruded four inches, livid and excoriated in several places. Having returned it with some difficulty, an assistant retained it by pressing his finger firmly upon one margin of the anus, while I clipped out with the scissors five of the radiating folds of skin, the incisions being carried a little over the inner margin of the anus. There was no hemorrhage of importance, and such was the stimulus given to the sphincter by the cuts, that the bowel was immediately kept up without pressure. For this operation, as beautiful as it is simple, we are indebted to the genius of the celebrated Dupuytren. It is proper to state, that, prior to the operation, all the usual means of relief, such as a compress retained by a T bandage, astringent injections, &c., had been resorted to, but without success.

*Variola and Vaccine.*—Small pox prevailed in this city to a considerable extent during the first three months of this year. In the house where I saw my first case, was a mother with an infant four months old, both unprotected by vaccination. I vaccinated them with some very active virus, and on the eighth day, when the vesicle was fully formed, I directed them both to be carried into the room where the patient with small pox was lying, then the eighth day from the appearance of the eruption. From that day, both mother and child were freely exposed to the infection, and both escaped without the slightest indisposition. The grandfather of the infant, who had been inoculated thirty years before, and had the variolous disease fully, as shown by marks upon his person, had varioloid severely, as did five of his children who had had varioloid some years before, not one in the family escaping the disease except the grandmother and the mother and child before mentioned. This would seem to prove that *fresh* vaccination is a better prophylactic than *old* small pox.

My second case of variola occurred in the person of a negro, who was seized with a chill on Sunday, and broke out on the following Wednesday. I saw her for the first time on Friday ; the eruption was then just passing from the papular into the vesicular stage. Her child, three years old, had been sleeping with her from the beginning of her sickness. I immediately vaccinated it upon the left arm in two places. Ten days after, and when the mother was convalescing, there was no trace of the punctures upon the child's arm. I vaccinated her again in the right arm. A week after this the child was seized with all the symptoms of small pox Upon examining the arms, I found that upon the *left* one were two vaccine vesicles. The case turned out to be one of very mild varioloid.

I saw another case, in the practice of Dr. Tunstall, which had been very freely exposed to the variolous infection, and in whose system the vaccine virus lingered for fourteen days without manifesting itself locally. It at length made its appearance simultaneously with the variolous disease, and seemed so to modify it as to make the case one of mild varioloid.

I treated all the cases I had under my care with a purgative, now and then, of calomel and rhubarb, and a solution of nitrate of potassa during the high febrile stage. The fact frequently mentioned in the books, particularly struck me, viz : that the initiatory symptoms of varioloid are fully as severe as those of confluent small pox.

Since the 15th of January, I have vaccinated 179 persons, of which 40 were *re*-vaccinations. Of these last, 34, or about 75 per cent., took the disease a second time. The susceptibility in some cases to the disease, a second and even a third time, is singular. In 1846 I vaccinated a little girl, with success—in '47 repeated the operation, with the same result—in '48 vacccinated her again, and she again took the disease. Last spring, upon trying it again, no impression was produced even locally. She has a brother and a sister, who have each had the vaccine disease perfectly, twice. The scars from the last operation are just as distinct and characteristic as from the first.

*Presentation of the Shoulder.*—April 16th, 1849, was called to see a negro woman, about two miles from the city. She had been in labor since the 10th, although the pains were not active until 48 hours before I saw her. The waters were discharged on the 15th, in the morning. The patient was 20 years old, well formed, and had had one child. Upon making an examination, I found the hand and arm in the vagina, and the os uteri considerably dilated, soft and yielding; pains feeble and irregular. Having determined upon version by the feet, the bladder and rectum being emptied, I bled her to sixteen ounces, and administered 80 drops of Tr. Opii. I passed my hand readily into the uterus, and in so doing carried up the hand of the child with it. It was the right arm that was down, and the head was in the left iliac fossa, the face being towards the back of the mother. I found the feet without difficulty, but the waters had been discharged so long, and the tonic contraction of the uterus was so great, that I hesitated to perform the evolution, for fear of doing irreparable injury to that organ. Under these circumstances, I determined to try version by the vertex. The arm being already returned into the uterus, I could more easily seize the vertex, which I succeeded in doing, and brought it into the axis of the superior strait in the first position. I then withdrew my hand, and the pains having ceased almost entirely, I left the patient for two hours. Upon my return, found more active contractions, and the chord prolapsed. Attempted several times to return it, but in vain, for the head pressed too firmly against the pelvic brim to allow it to pass. The child was delivered in four hours from the time I returned. The labor was protracted by very great posterior obliquity of the uterus, which threw the head of the child strongly against the pubis. This was obviated, in some degree, by pressing firmly upon the hypogastrium during each pain.

I observed another difficulty in this case, which I had never met with before, and which, though seemingly a trifle, embarrassed me a good deal: when the hand was introduced into the vagina, there was produced in the palm so complete a vacuum, that I found it next to impossible to separate my thumb from the fingers; nor could I get along at all, until I let the air in with the forefinger of my left hand. The child, when born, had been dead about ten hours; the mother did well.

*Hour-glass Contraction and retained Placenta.*—Mrs. S., æt. 30, was taken in labor with her first child, April 26th, the pains being very slight until the 28th. Waters were discharged on the 27th. First position of the vertex, and the child was delivered the next day at 5 o'clock, P. M. Waited 20 minutes for the expulsion of the placenta, but finding little or no effort to throw it off, I introduced my hand for the purpose of bringing it away, having first made gentle traction upon the chord. I was distressed to find a complete hour glass contraction of the uterus, and the placenta shut in above the constriction. Having placed my fingers together in a conical form, I slowly and cautiously proceeded to overcome the difficulty, which required both time and patience. My hand at length passed into the upper chamber, when I found the placenta adherent to the fundus uteri. It was detached with great difficulty, being peeled off with the fingers just as the butcher peels the leaf-fat from the inside of the hog. As the placenta came away, the uterus contracted firmly. I had frequently seen irregular contractions of the uterus, but never before a perfect hour-glass contraction. This state of that organ has by many been considered as impossible.

(To be concluded in next No.)

